# WISCOD: A Statistical Web-Enabled Tool for the Identification of Significant Protein Coding Regions

**DOI:** 10.1155/2014/282343

**Published:** 2014-09-15

**Authors:** Mireia Vilardell, Genis Parra, Sergi Civit

**Affiliations:** ^1^Department of Vertebrate Genomics, Max Planck Institute for Molecular Genetics, Ihnestraße 63–73, 14195 Berlin, Germany; ^2^Department of Evolutionary Genetics, Max Planck Institute for Evolutionary Anthropology, 04103 Leipzig, Germany; ^3^Department of Statistics, University of Barcelona, Avenida Diagonal 643, 08028 Barcelona, Spain

## Abstract

Classically, gene prediction programs are based on detecting signals such as boundary sites (splice sites, starts, and stops) and coding regions in the DNA sequence in order to build potential exons and join them into a gene structure. Although nowadays it is possible to improve their performance with additional information from related species or/and cDNA databases, further improvement at any step could help to obtain better predictions. Here, we present WISCOD, a web-enabled tool for the identification of significant protein coding regions, a novel software tool that tackles the exon prediction problem in eukaryotic genomes. WISCOD has the capacity to detect real exons from large lists of potential exons, and it provides an easy way to use global *P* value called expected probability of being a false exon (EPFE) that is useful for ranking potential exons in a probabilistic framework, without additional computational costs. The advantage of our approach is that it significantly increases the specificity and sensitivity (both between 80% and 90%) in comparison to other ab initio methods (where they are in the range of 70–75%). WISCOD is written in JAVA and R and is available to download and to run in a local mode on Linux and Windows platforms.

## 1. Introduction

Identifying protein-coding sequences is the first and one of the most important tasks after sequencing a genome. To date, over 340 eukaryotic genome sequencing projects have resulted in genome assemblies [1]. It is crucial to have reliable tools for the automatic annotation of any given DNA sequence of an organism in which the primary DNA structures are not currently known, including the number and location of genes, the location of exons and introns (in eukaryotes), and their exact boundaries [2]. However, the task of deciphering the gene structure from uncharacterized DNA sequences is not trivial and computational gene recognition programs are a critical part of their automatic annotation [3].

From the beginning of genomic sequencing, an established goal has been provided to give accurate* ab initio* prediction of genes, that is, the identification of gene structure using only information inherent in the DNA sequence. The problem remains largely unsolved for eukaryotic genomes where coding elements are substantially punctuated with introns. Determining methods for* ab initio* gene detection has been an active area of research and a number of gene prediction algorithms have been developed [3]. Generally, they are based on detecting boundary signals (*starts* and* stops*) and splicing sites (*acceptors* and* donors*) as well as the* coding region* for each exon (exon components). Recent reviews of* ab initio* methods indicate that they can correctly predict more than 90% of the coding bases and 70–75% of exons. However, correct gene structure prediction falls to 50% [3, 4]. To avoid this loss of accuracy, new algorithms that take advantage of information from other sources have been developed. For example, predictions are conducted based on similarities between the query sequence and the manual annotation of genes or from full-length cDNA databases (e.g., [4]). Other algorithms exploit sequence conservation among species to determine the position of new potential genes [3, 4]. In other approaches, the output of two or more gene predictors is combined for improved performance (e.g., [5, 6]) which can increase exon recognition to as much as 85% [3, 4, 7]. However, it is generally acknowledged that improvement at any step of the process could result in better performance of global gene prediction.

Here, we present a method, integrated in multiplatform Java-R open software called WISCOD, which is useful to rank exons on the basis of a probabilistic framework without computational costs. After imputing *P* values for each exon component (usually three *P* values, one for each boundary and one for the coding region), the method combines them according to meta-analysis statistical methodology (MA tests) [8] and assigns only one expected probability of being a false exon (EPFE values) to each potential exon tested. EPFE values are easily interpreted: potential exons with lower values are more likely to be genuine exons.

Thus, potential exons (usually ranging from 10,000 to 30,000 for a potential gene) can be sorted by minimal EPFE value to reduce drastically their number (to approximately 300–1,500) with only a minor loss of the real ones (sensitivity and specificity 85%). EPFE values are based on probabilistic methods free of bias like exon type or exon length, making them easier to interpret. Additionally they have appeared more robust among different conditions than actual methods. We also provide a false discovery rate control procedure [9], a well-known statistical methodology used in multiple hypotheses testing to correct for multiple comparisons. In this work, we compare our method with that of one of the most important gene prediction programs [10] and others [11] (Supplementary data, Section 5). Additionally, we test our method in three different scenarios as follows:

1. WISCOD performance and its comparison with the sum scores tests (SST) from GeneID [10],
2. MA tests and EPFE values, a powerful WISCOD module to rank exons: an example using the HAVANA project data [12], and
3. WISCOD application: an example using PAX5 gene chromosome 9p transcripts.

## 2. Material and Methods

The WISCOD system consists of two modular parts, called* Model testing* and* Exon testing*, which are connected together via a front-end web interface system. The results can mostly be retrieved and displayed textually or graphically.* Model testing* assigns *P* values to each exon component using a simulation approach and tests* ab initio* models.* Exon testing* assigns EPFE values to each exon based on the *P* values for each exon component; these EPFE values can then be used to rank exons by their probabilistic framework.

WISCOD is written in Java (http://www.java.com/) and R (http://cran.r-project.org/ [12]) programming languages. It is freely distributed and continuously improved. The compiled code can be run under different operating systems (Windows, Linux, Mac, or any platform supporting Java Runtime Environment version 1.5.0 or higher and R version 2.7 or higher). The minimum estimated hardware requirements are a Pentium III processor and 256 MB of memory.

### 2.1. Model Testing Module: A Simulation Approach to Assigning *P* Values to Exon Components

Classically,* ab initio* methods are based on scoring potential exon component sequences given two previously predefined models. One of the models is called the background model (Q) which contains the expected frequencies for noninformative sequences or false exons; the other is called the real model (P) and contains the expected frequencies for informative sequences or real exons. From the scores, different types of scoring matrices (W) can be built in order to score new sequences and classify them as false (low score) or real (high score) [10, 13, 14]. The scoring system used in this paper was derived from natural logarithm ratios of the expected real frequencies (P) versus the expected background frequencies (Q) [10].

In previous work, [15] developed a simulation method that allows *P* values for the boundary signals to be assigned and for the coding region of an exon given a Q model and a score matrix W. Note that not only the Q model is used to input the *P* values but also the P model is necessary to score the sequences (see Supplementary data, Section 2). Therefore, this module also allows the goodness of fit of both proposed models, Q or P, to be checked.

#### 2.1.1. Model Testing Module: Input Description

In this module the users assign *P* values by simulation to a set of their sequences either by using predefined models (*Drosophila melanogaster* or* Homo sapiens*) or by uploading their own models that basically consist of uploading the probabilities under the Q model, probabilities under the P model, and the score matrix, W.

**Figure 1:**
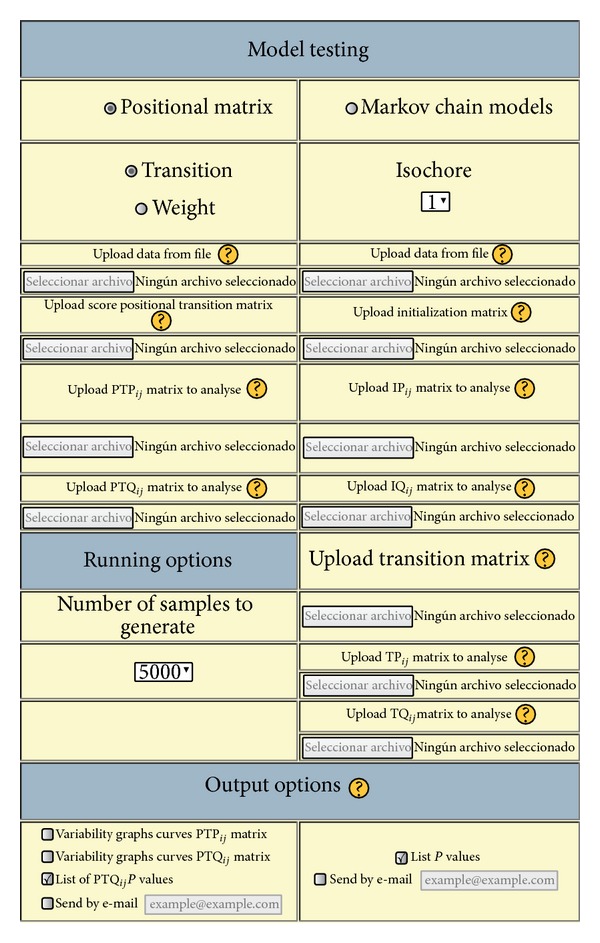
WISCOD: model testing module.

Figure 1 shows the* model testing module* options which are split into two parts:

1. *positional models* including positional weight matrices (PWM) and positional transition matrices (PTM);
2. *Markov chain models* (MCM).

Basically, all of the options rely on the same idea, that is, the generation of sequences under the null hypothesis (here, defined by the frequency matrices of the Q model) which are scored by the score matrix, W. Finally, a *P* value based on the quantiles of this empirical distribution generated by sequence simulation is assigned to each test sequence.

Briefly,* positional models* are defined by a set of expected frequencies and scores for every nucleotide base at every sequence position. When these frequencies do not depend on previous nucleotides, the models are called* PWM*; otherwise they are called* PTM*.

The main difference between the positional models and the* Markov chain models* is the fact that the scores and probabilities of the latter do not depend on the position, only on the nucleotide history of the sequence. In this case, it is necessary to upload a weight matrix (initialization matrix) which contains the scores for the first block of nucleotides, as well as the probabilities in the real model (IP) and the probabilities expected in the random or background model (IQ). It is also necessary to upload a transition matrix to score the following nucleotides as well as the probabilities in the real model (TP) and the expected probabilities in the random or background model (TQ). Examples and more information about the matrix formats are available in the WISCOD help.

#### 2.1.2. Model Testing Module: Output Description

The usual WISCOD output for this module is a list of *P* values for the input sequences (exon components). WISCOD also provides a graphic interface to test the goodness of fit of the proposed PWM or PTM models which requires previous knowledge of the model of the sequences provided by the user (this means whether the sequences belong to the background model, Q, or the real one, P). Basically, a statistical comparison is performed between the score distribution of the input sequences and the score distribution derived from the proposed models. Then the user can test how similar their input data are to the predefined models. Usually this comparison is performed via the Kolmogorov-Smirnov test. However, it is known that tests could become significant even with small differences when a large amount of data is used; so we have decided to rely on graphical methods.

**Figure 2:**
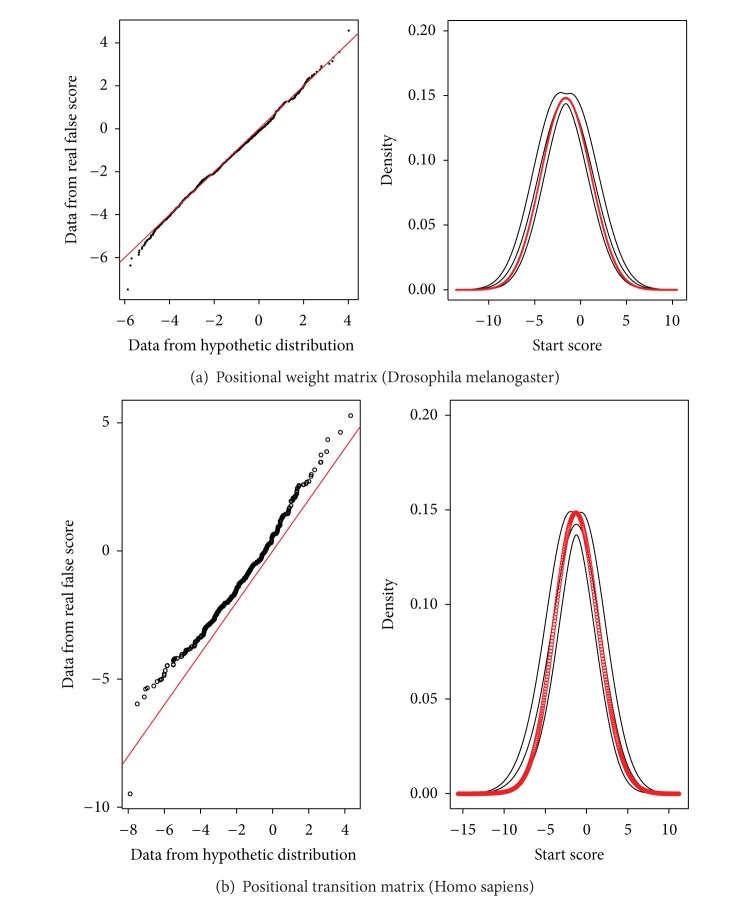
Model testing module output.

- Probability plots have the scores obtained from sequences generated by simulation along the *X*-axis and the corresponding scores obtained from sequences provided by the users on the *Y*-axis (Figure 2).
- Variability graphs, which are not implemented as standard in statistical packages, draw the nonparametric density of the input sequences when their model is known (real or false) and an error bar obtained by the simulation of sequences under the specified model (Figure 2).

Figure 2 shows an appropriate goodness of fit from two real situations: one from a PWM and the other from a PTM (here, equally distributed nucleotides are considered to manage the dependency in the initial positions).

### 2.2. Exon Testing Module: A Tool to Rank Potential Exons

This module implements tests in order to rank exons by using a probabilistic framework, which is primarily based on combining exon component *P* values (by applying the intersection-union test, IUT [16], or the MA approach [8]) and, additionally, the GeneID score system is also implemented by the sum of scores test (SST) [10]; a more detailed description can be found in the Supplementary data, Section 1.

#### 2.2.1. Exon Testing Module: Input Description

**Figure 3:**
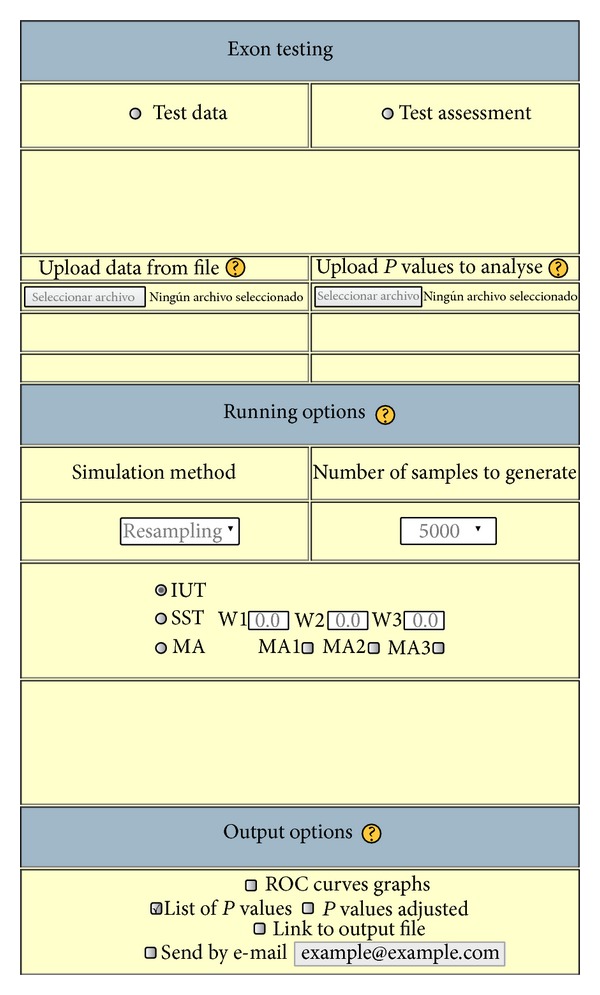
WISCOD: Exon testing module.

Figure 3 shows the* exon testing module* which is composed of two parts:

1. *test data* refers to the assigned probability measure when sequences from* Drosophila melanogaster* or* Homo sapiens* are provided;
2. *test assessment* refers to the assigned probability measure to a list of potential exons which are mainly characterized by three *P* values (one for each exon component). The latter are* previously* obtained by the* model testing module* in a collaborative way.

**Figure 4:**
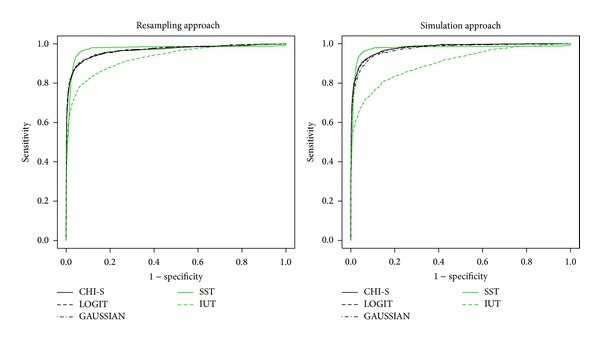
ROC curves by simulation and estimation methods.

The* test data* option also offers the possibility of inferring *P* values from each exon component using alternatives to the simulation approach, that is, by resampling and by estimation (see Supplementary data, Section 2). Resampling and simulation methods could result to slow, in particular, assigning *P* values to the coding region. To solve this problem, WISCOD uses a unique large simulation distribution for each site/organism (*N* = 10, 000) and a theoretical approximation based on a normal distribution (for more details, see Supplementary data, Section 2) to input *P* values for the coding region (Figure 4).

#### 2.2.2. Exon Testing Module: Output Description

WISCOD outputs result in text *P values*,* adjusted* *P values* [9], and graphical ROC curves. When the model underlying each sequence is known, then it is possible to display ROC curves using the WISCOD exon testing module. ROC curves measure the general appropriateness of all tests according to* S*pecificity and* S*ensitivity values. Figure 4 shows ROC curve differences using the simulation and estimation approaches. Our results show no differences between the two.

## 3. Results and Discussion

We demonstrate the utility of WISCOD by analyzing three different datasets for two different organisms: one for* Drosophila melanogaster* [10, 15] and the other for* Homo sapiens* (for more details, see Supplementary data, Section 4).

### 3.1. WISCOD Performance and Its Comparison with the Sum Scores Test (SST) from GeneID

Several studies have been published on the* exon length bias problem* [17, 18] and nowadays gene prediction programs such as [19] the use of this information to make more accurate exon predictions. Using the WISCOD exon testing module, we reanalyzed this problem according to the following variables:

- *species: Drosophila melanogaster* and* Homo sapiens*,
- *exon types*: first, internal, terminal, and single,
- *GC* content as well for* Homo sapiens* by defining three different isochores (GC content) (for more details, see Supplementary data, Section 4).

In the case of a binary predictor diagnostic test, performance can be evaluated using the measures of sensitivity and specificity. However, in many instances, we encounter predictors that are measured on a scale or continuum. In such cases, it is desirable to assess the performance of the diagnostic test over the range of possible cut-off points for the predictor variable. To achieve this, a receiver operating characteristic (ROC) curve is used that includes all the possible decision thresholds for the diagnostic test result. In this brief report, we discuss the salient features of ROC curves. We also discuss and interpret the area under the ROC curve and its utility in comparing two different tests or predictor variables of interest. In [15] MA methods are extensively compared with the SST [10].

**Figure 5:**
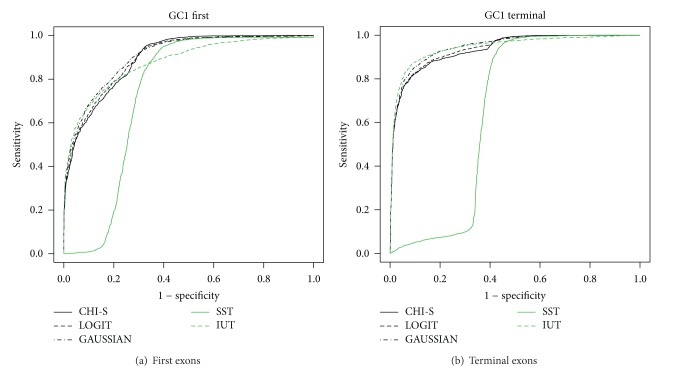
GC1* Homo sapiens* ROC curves.

**Figure 6:**
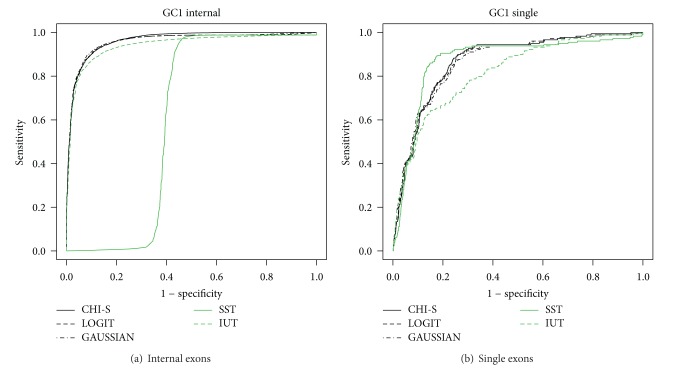
GC1* Homo sapiens* ROC curves.

Only for illustrative purposes,* e*xon types for* GC1 Homo sapiens* are shown in Figures 5 and 6. The results show the performance of the SST [10], IUT, and MA tests (described in Supplementary data, Section 3) using ROC curves. The ROC curves show in all the scenarios (regarding species, exon type, and GC content) that the MA methods are more robust than the others.

The MA tests have a sensitivity and specificity of between 80% and 90% in all the scenarios while for the SST and IUT tests this can drop significantly (Figures 5 and 6). Here, we should remark that the tests based on *P* values (IUT and MA) take into account the exon length bias directly by assigning a coding region *P* value with regard to the length meaning that the coding region distribution is built from scored sequences with the same length as those tested (see Supplementary data, Section 3). This means that exons with different lengths can be directly compared using IUT or MA. However, the SST scores are based on the sum of the scores obtained for each position or base pair. This means that large coding regions have more summands than short ones and this could produce an exon length bias. Otherwise, Figures 5 and 6 show that methods based on MA (CHI-S, LOGIT, and GAUSSIAN) are more robust when faced with the exon length problem.

**Figure 7:**
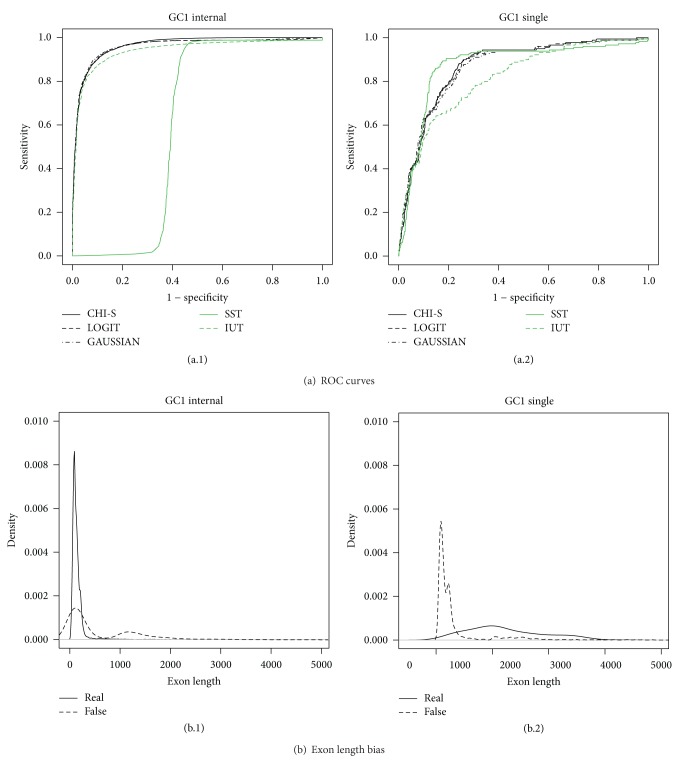
Exon length bias problem.

In addition, Figure 7 shows the improvement our method represents when tackling the exon prediction problem in* Homo sapiens*. Globally the figure shows

- a decrease in the overall performance of the SST method (Figure 7(a.1)) when the exon length distributions between real and false exons are close (Figure 7(b.1));
- in contrast, a globally good performance of the SST method (Figure 7(a.2)) in single exons, due to a clear differential exon length distribution (Figure 7(b.2)) between real and false exons.

Thus, due to their better performance and robustness, we strongly recommended the MA methods to rank exons.

### 3.2. MA Tests and EPFE: A Powerful WISCOD Tool to Rank Exons—An Example Using the HAVANA Project Data

A validation study was conducted with the second* Homo sapiens* dataset concerning the capacity of MA methods to rank exon structures. In all cases, potential exons were extracted using the GeneID program version 1.3.15.

The false sets of exons used in the previous section were built using false coding regions flanked by false site sequences. Therefore, it is necessary to validate our method in a real situation where the false potential exons share their coding region and/or real sites with real potential exons. So, here we validate the sensitivity and specificity of the MA tests using a* Homo sapiens* HAVANA dataset taking into account the situation of having partial real exons. A first step in solving such a problem is to acknowledge its dimension.  Table 1 shows the magnitude of the problem to deduce real exons which account for a very low percentage, around 0.10%, of the total list of candidates (on average, each list contained 26,650 potential exons).

In summary, Table 1 shows the performance of the MA tests for a given EPFE value cut-off.

1. The best values of sensitivity (expressed here as the % of real exons detected (around 80–87% on average and greater than 95% as a median) are given for EPFE values ranging between 0.005 and 0.01,
2. Keeping a reasonably high degree of specificity (on average the exon candidates consist of a few hundred, compared to the huge total number, which is greater than 25,000) is also given.

In this section we do not show the SST because it does not have a clear threshold and may not be directly comparable between exon types.

Additionally, we found that the three MA methods (CHI-S, LOGIT, and GAUSSIAN) perform fairly equally.

Furthermore, we have used HAVANA data to test other leading gene identification programs. For illustrative purposes we show the results for [11] (Supplementary data, Section 5). As for other gene identification programs, the sensitivity values provided by AUGUSTUS depend on the exon type considered; they range from 19% to 80% for single combinations and from 19% to 86% for multiple combinations (Table 2). According to this, our method is shown to be more robust.

### 3.3. PAX5 Gene Chromosome 9p WISCOD Application

The fact that our method detects exons without making any assumption regarding the consequent gene structure can be used to deduce, simply, the encrypted exon(s) [20] in a DNA sequence query which can take part of different transcripts from the same gene [21].

**Figure 8:**
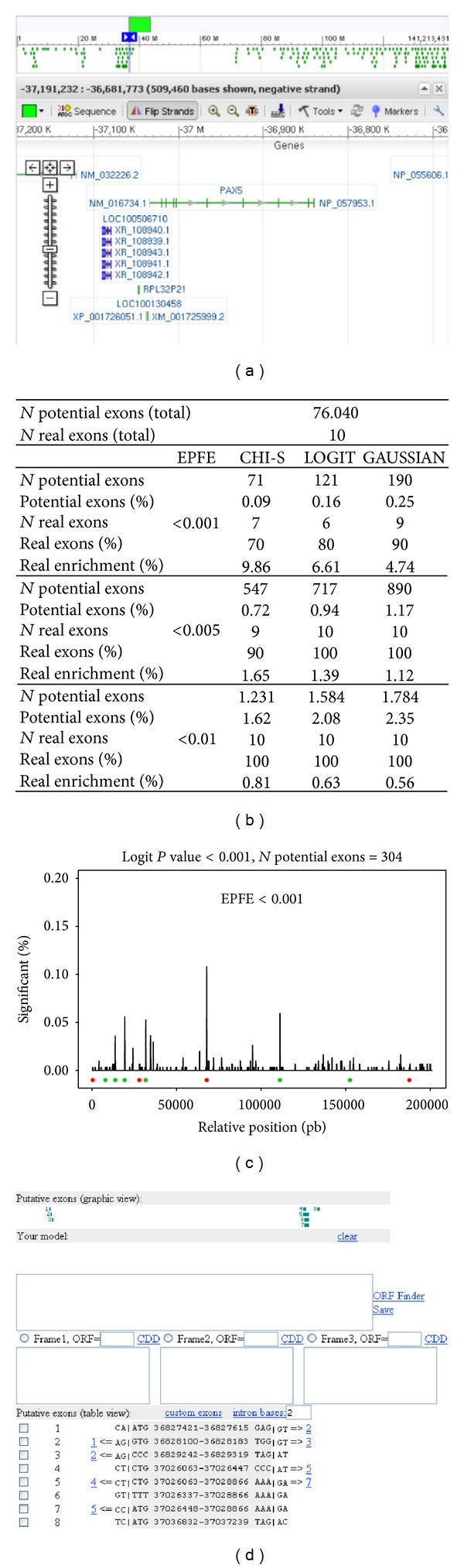
PAX5 GENE. (a) PAX5 location, (b) using WISCOD to deduce PAX5 exons, and (c) peaks show enrichment of significant exons at some DNA positions and circles represent real exons, which are green if they were detected at this EPFE value and red if not. (d) Model marker program.

In this section, we tried to identify the known exons of the PAX5 gene which is located on chromosome 9p with an inverse orientation (Figure 8(a)). The PAX5 gene is composed of 10 different protein-coding exons (identified using the Ensembl database [22]) with the classical boundaries [23]. A GeneID search of the DNA sequence allowed using a potential list of exons that contained *N* = 76, 040 potential structures, meaning that only 0.02% are real exons. With EPFE values below 0.005, we were able to detect all the potential coding exons (Figure 8(b)) and to reduce significantly (i.e, by 98.6% with the logit method) the list of potential exons. Since some nonreal potential exons can share a coding region and/or boundary signals, it is difficult to get a precise division of the two groups (real versus false). However, and because of that, real exons have a tendency to include neighboring or to be inside high density regions of potential exons with small EPFE values (Figure 8(c)). So, we used this information to detect the real ones.

Additionally, our output could be combined with other sources of evidences; that is, Figure 8(d) shows the model marker [24] input (http://www.ncbi.nlm.nih.gov/mapview/). This software offers the possibility to build exon or gene structures using additional user information (our model section, see Figure 8(d)) or by selecting the most plausible ones from a list of candidates provided by model marker (putative exons section, see Figure 8(d)).

## 4. Conclusion

With a large number of sequence data becoming available, statistical analyses can be applied to these data and will offer beneficial output to research communities. In summary, we provide the bioinformatic and geneticist communities with an implemented statistical and graphical tool that allows them to compare and evaluate mathematical models used in* ab initio* methods to predict primarily DNA structures and also software that can be used to prioritize exon candidates given a DNA sequence with only a minor loss of information by reducing the number of potential exons drastically (>90%). Prioritization comes through minimal EPFE values which agglomerate different types of evidence for an exon in a single value.

Our methodology based on MA tests is more robust and can directly compare outputs. That is in contrast to the SST for which, in the first step, splice sites and start and stop codons are predicted and scored along the sequence using different position weight matrices (PWMs). Our methodology is also free from exon length bias and works better in a large range of situations, as well as being easy to use because the* MA approach* provides a unique probability: the* EPFE values*. The interpretation of the* EPFE values* is easy: the lower values correspond to more suitable options. We also provide an FDR control (BY) procedure.

Our methodology is capable of differentiating real exon structures from false ones with a sensitivity and specificity close to 85% in two different organisms (*Drosophila melanogaster* and* Homo sapiens*), even when only a small fraction of exons are present (<0.1% of the total potential ones). This means that there is a clear improvement in tackling the exon detection problem compared to other* ab initio* programs (>10%) and similar performance values are achieved to those of strategies based on combining more than one algorithm.

Moreover, in order to make exon structures more plausible, it is possible either to combine the WISCOD output with other software (i.e., model marker) or to introduce more sources of evidence as *P* values in the EPFE values simply by modifying the degrees of freedom in our MA system.

Finally, many gene prediction programs such as AUGUSTUS provide filtered lists of exons whose scores are hard to interpret in a probabilistic or statistical way. We therefore provide this application, which displays exons and ranks them on the basis of a probabilistic framework, in the hope of filling a gap in the toolbox currently available to researchers.

To make our tool available to the research community, WISCOD can be downloaded and run in a local mode.

## 5. Supplementary Information

WISCOD is available at https://www.dropbox.com/sh/oq7tl64l15v2z5d/Mfhb_bhvNz/wiscod_windows.zip. https://www.dropbox.com/sh/oq7tl64l15v2z5d/0AlES9y04E/wiscod_ubuntu.zip.

## Figures and Tables

**Table 1:** The *capacity* to detect real exons from a large list of potential exons taking account of different levels of confidence (EPFE values) in a HAVANA subset of manual curated genes on HSA21.

*N* transcripts = 116		Mean			Med.			q25			q75		
*N* potential exons (total)		26649.59			10790.00			3870.50			33168.00		
*N* real exons (total)		9.23			7.00			3.00			13.00		
% real enrichment		0.11			0.08			0.03			0.14		

	EPFE	CHI-S				LOGIT				GAUSSIAN			
		Mean	Med.	q25	q75	Mean	Med.	q25	q75	Mean	Med.	q25	q75

*N* potential exons		39	27	9	55	68	46	17	89	110	77	23	141
**% real exons**	**<0.001**	**53.9**	**61.3**	**33.3**	**80.0**	**64.7**	**72.7**	**50.0**	**91.8**	**70.1**	**85.7**	**50.0**	**100**
% real enrichment		16.9	16.3	6.0	22.4	12.0	11.1	4.4	14.6	8.5	7.8	3.2	11.6

*N* potential exons		300	201	58	376	387	252	75	483	473	305	88	587
**% real exons**	**<0.005**	**79.6**	**96.2**	**82.5**	**100**	**81.4**	**100**	**83.3**	**100**	**81.9**	**100**	**82.5**	**100**
% real enrichment		3.9	3.4	1.3	5.9	3.1	2.6	1.0	4.4	2.7	2.4	0.8	3.8

*N* potential exons		604	369	105	725	788	493	134	918	857	537	143	1002
**% real exons**	**<0.01**	**84.9**	**100**	**87.1**	**100**	**85.5**	**100**	**88.9**	**100**	**86.3**	**100.0**	**90.7**	**100**
% real enrichment		2.2	1.9	0.7	3.1	1.8	1.5	0.6	2.6	1.7	1.5	0.6	2.5

*N* potential exons		1291	774	206	1465	1511	852	240	1697	1596	887	252	1795
**% real exons**	**<0.02**	**89.3**	**100**	**100**	**100**	**90.5**	**100**	**100**	**100**	**89.4**	**100**	**100**	**100**
% real enrichment		1.2	1.0	0.4	1.7	1.1	1.0	0.3	1.6	1.0	0.9	0.3	1.5

**Table 2:** Augustus prediction.

*N* real transcripts = 116									
*N* exons = 1087									
		Number of real predictions		Sensitivity		Total number of predictions		Real enrichment	
	Real exon content	Single prediction	Multiple prediction	Single prediction	Multiple prediction	Single prediction	Multiple prediction	Single prediction	Multiple prediction

Start	67	26	29	39%	43%	100	179	26%	16%
Internal	925	743	796	80%	86%	1031	1316	72%	60%
Terminal	79	43	46	54%	58%	103	157	42%	29%
Single	16	3	3	19%	19%	9	10	33%	30%

Total	1087	815	874	75%	80%	1243	1662	66%	53%
